# Landscapes of care and despair for rural youth – a qualitative study in the northern Swedish ‘periphery’

**DOI:** 10.1186/s12939-020-01288-z

**Published:** 2020-10-02

**Authors:** Frida Jonsson, Isabel Goicolea, Monica Christianson, Dean B. Carson, Maria Wiklund

**Affiliations:** 1 Department of Epidemiology and Global Health, Umeå University, Umeå, Sweden; 2 Arctic Research Centre (Arcum), Umeå University, Umeå, Sweden; 3 Department of Nursing, Umeå University, Umeå, Sweden; 4 School of Business and Law, CQUniversity, Rockhampton, Australia; 5 Department of Community Medicine and Rehabilitation, Unit of Physiotherapy, Umeå University, Umeå, Sweden

**Keywords:** Northern Sweden, Rural, Youth, Health, Landscapes of care, Thematic analysis

## Abstract

**Background:**

This study emerges as a response to the lack of youth perspectives when it comes to discussions about access to and experiences of health and social services in rural areas. It subsequently contributes to the literature by positioning young people at the centre of this debate, and by taking a more holistic approach to the topic than is typically the case. Specifically, based on the idea that a good life in proper health for young people may be contingent on notions of care that are bounded up in multi-layered social and spatial environments, the aim of this study was to explore what characterises ‘landscapes of care’ for rural youth.

**Methods:**

In this qualitative study, the participants included young people and professionals residing in five diverse areas across the northern Swedish ‘peripheral’ inland. Individual interviews (16 in total) and focus group discussions (26 in total) were conducted with 63 youth aged 14–27 years and with 44 professionals operating across sectors such as health centres, school health, integration units, youth clinics and youth clubs. Following an emergent design and using thematic analysis, we developed one main theme, ‘landscapes of care and despair’, comprising the two themes: ‘(dis)connectedness’ and ‘extended support or troubling gaps’.

**Results:**

The findings illustrate how various health-promoting and potentially harmful aspects acting at structural, organisational and interpersonal levels contributed to dynamic landscapes characterised simultaneously by care and despair. In particular, our study shows how rural youths’ feelings of belongingness to people and places coupled with opportunities to participate in society and access practical and emotional support appear to facilitate their care within rural settings. However, although the results indicate that some in the diverse group of rural youth were cared *for* and *about*, a negative picture was painted in parallel. These aspects of despair included youths’ senses of exclusion and marginalisation, degrading attitudes towards them and their problems, as well as recurrent gaps in the provision and practices of care.

**Conclusions:**

To gain a more comprehensive understanding about the health of rural youth, this study highlights the benefits investigating ‘care-ful’ and ‘uncaring’ aspects bounded up in dynamic and multi-layered landscapes.

## Background

Over the last few decades, rural communities in the global north have gained attention from researchers in a variety of fields [1]. This focus builds on a wish to understand the concept of rurality, but emerges also from international [2] and Swedish [3] concerns about the challenges facing ‘peripheral’ areas characterised by remoteness, low population densities and marginalisation. Based on this notion, studies have described ‘the rural’ as a spatial and social location for and representations of a good life, while also depicting these areas perceived inferiority [4–6]. Such notions are representative of broader tendencies within public, political and scientific realms, where rural aspects including health are evaluated largely for how they compare to their urban counterparts [7–9]. They also refer to general foci on the problems faced by, rather than the strengths of, rural people and places as well as to the fact that rural demographic and structural change is often constructed as processes of deficit and decline, despite the picture generally being more complex [10].

One of the growing challenges in the rural north is the depletion of welfare resources from peripheral communities [11, 12], where shrinking and ageing populations place increased pressure on systems that, for example, continue to find it difficult to recruit and retain skilled and professional workers [13]. However, with notable exceptions of (contested) expectations about youth mobility and migration [14–17], research striving to improve our understanding about what is happening to, and going on in, rural areas has so far been both adult- and elderly-centric, thus largely failing to account for the diverse perspectives of youth [18, 19]. To help bridge this knowledge gap, the purpose of this research was to situate young people at the centre of debates about access to and experiences of rural health and social services, while adding to the literature by allowing rurality to ‘stand apart’ from urban comparison, and by considering a more holistic view on the issue than is typically depicted.

In this regard, we follow recent discussions within the public health literature (see, for example, [20]) where Leonardi move beyond the construction of health as a static state of “complete wellbeing” ([21] p. 736), to an understanding of the concept as an ongoing, iterative process involving opportunities and capabilities to satisfy needs, realise aspirations and cope with various situations. From this point of view, health represents a valued end in itself, in addition to being a mean that allow people and groups to participate in society [20]. Acknowledging this shift thus highlights the importance of promoting the health of rural youth. At the same time, it illustrates that the ways through which this could be achieved might not be limited to the delivery of health and social services, but include notions of care involving experiences bounded up in multi-layered environments or landscapes [22].

By integrating the heterogeneous perspectives of young people and professionals living in five diverse areas across the northern Swedish peripheral inland, the aim of this study was to explore what characterises ‘landscapes of care’ for rural youth.

### Conceptual framework

Guided by the richness of our data following an emergent design (see analysis section below) and the belief that a good life in proper health for rural youth might extend beyond the availability of or access to services, we utilise the concept ‘landscapes of care’ [23] as an analytical lens. Specifically, to understand more fully what aspects are important to ensure rural youth a healthy life, we situate young peoples and professionals experiences within a framework that allows us to capture not only what health and social services are provided, but how they are delivered and perceived (using the concept of care), and in which contexts and circumstances (through to the concept of landscapes).

In this regard, we follow Milligan and Wiles [23] by considering ‘landscapes of care’ to be a multi-layered concept that encompass acts and affective dimensions at interpersonal levels; arrangements and accountabilities at organisational levels; and policies, discourses and norms at structural levels. Jointly, these aspects are then presumed to shape the experiences, delivery and practices of care within and across landscapes that, in line with Bell and colleagues [24], represents a symbolic space of familiarity and culture while being characterised by physical features and social conditions. In this context, care refers to the provision or receipt of emotional and practical support [23]. This means that it comprises affective dimensions of embodied subjectivities, ongoing commitments of seeing and responding to needs, as well as interdependencies between acts and actors involved in the mutual co-production of care [25–27]. From this perspective, it may be conceptually helpful (albeit not always desirable or feasible) to discern between different realms of care. Milligan and Wiles [23] thus distinguishes between performances of care-giving from emotional aspects of care utilising the concepts of caring *for* and caring *about.* With the idea of caring *for*, we recognise that care involves more formal activities and actions undertaken by institutions and/or professionals. With the notion of caring *about*, in turn, we view care as an embodied phenomenon involving affective elements that emerge through subjective experiences of how body-space interactions make us think and feel [28].

Rather than considering care as a unidirectional activity, similar to Milligan and Wiles [23] we further believe that all involved parties may gain in ways that directly or indirectly improves their health. For example, defined as emotional (such as attentiveness, empathy and encouragement) and practical (as in the provision of services or practical help) support, care might not only make life easier for the recipients, but also make them feel better. In addition, by contributing to a sense of pride and reciprocal appreciation, care-giving/being caring might be empowering to those who perform it [29] while also being potentially oppressive and objectifying, especially for women [30]. From the recipients’ perspective, in turn, care may represent a shameful dependency while it should rather be seen an essential aspect that ties people together [25]. As reflected in the neighbouring concept of ‘therapeutic landscapes’ [24], landscapes also appear to have health-promoting qualities, for example, through their pristine nature and possibilities to nurture experiences of relatedness, empathic interactions and healing senses of place.

Combined, the aforementioned ideas and developments make care an important yet still largely marginalised concern [31]. To the best of our knowledge, ‘landscapes of care’ have so far been used with reference to the deinstitutionalisation of services for different populations such as the elderly and the disabled [32, 33]. The limited application of this concept to understand what type of services and support are important and why to meet the multiple and diverse needs of young people in the global north more generally, and rural youth in particular, comprise an important gap worth bridging.

## Methodologies

### Study design and setting

In line with our explorative aim and intention to approach ‘reality’ as socially constructed, we designed the study around the qualitative methodology while using thematic analysis to scrutinize data collected through interviews and focus group discussions (FGDs). Additionally, as explained by Braun and Clarke [34], thematic analysis can be both inductive (data-driven) and deductive (analyst- or theory-driven); and in this research, we combine the two approaches following an emergent design. This study is also part of a larger research project studying the health of and care provided to young people living in rural northern Sweden [19].

Overall, the current study is situated in Sweden’s four northern-most counties: Norrbotten, Västerbotten, Västernorrland and Jämtland/Härjedalen. This area, popularly called *Norrland*, covers about 60% of the land area but in it inhabits approximately 12% of Sweden’s total population. With roughly five residents per square kilometre, this is a sparsely populated region where people live in rural villages in the inland or in somewhat larger cities along the coast. The area is home to the Sámi population comprising approximately 20–40,000 individuals [35] and a large number of international migrants, of whom many are unaccompanied children and youth [36].

#### Health and social service provision for rural youth

We focus specifically on the rural parts of Norrland’s interior where various institutions offer information, support and help to youth. The main health service providers are health centres, specialised child psychiatry units, school health and youth clinics, where the latter deliver services focused on sexual and reproductive health, in addition to medical and psychosocial support [37]. Beyond schools and formal health systems, social services such as youth clubs provide leisure activities and spaces for recreation, while youth councils facilitate socio-political participation. In addition, so-called (re) engagement initiatives help young people not in employment, education or training (“NEETs”) while municipal integration units support unaccompanied minors. Involvement and consultations are free of charge in all services. However, across northern Sweden in general, and the rural areas in particular, they are not evenly distributed which means that some areas lack amenities. This contributes to landscapes that vary largely by geographical location in terms of service provision, meaning that youth in some municipalities have greater access than others.

To capture the diversity in this region, five rural municipalities located in different parts of Norrland’s peripheral inland were included in the study. These sites were purposively selected according to their location, population size, socio-economic situation and availability of services for youths to attain contrasting and complementary perspectives (see Table 1 for features specific to each site).

**Table 1:** Site characteristics

	Site one (S1)	Site two (S2)	Site three (S3)	Site four (S4)	Site five (S5)
**Region**	A	B	C	B	D
**Population size**	5000–9000 inhabitants	< 5000 inhabitants	> 9000 inhabitants	> 9000 inhabitants	< 5000 inhabitants
**Distance to largest nearby city**	50–100 km	200–250 km	100–150 km	200–250 km	250–300 km
**Socio-economic situation**^a^	≈ 9%	≈ 6%	≈ 10%	≈ 4%	≈ 5%
**Facilities for youth**	Health centre School health (Re) engagement initiative Integration unit	Health centre School health Integration unit	Hospital Health centre School health Youth clinic Child psychiatry Youth club (Re) engagement initiative Youth council	Health centre Youth clinic Child psychiatry Youth club (Re) engagement initiative Youth council Integration unit	Health centre School health Integration unit

^a^Level of unemployment in the municipality for 2018

### Recruitment and participants

Participants were purposive selected for their ability to provide information that could contribute to address the aim of our project [19]. Initially, key-persons working with young people in diverse sectors (such as managers at health centres, youth clinics, school health and integration units) at each site were informed about the study via e-mail. We then visited each site to provide detailed information to potential participants and then met additional key-persons. In total, 44 professionals (33 women and 11 men) across the five municipalities participated in the study; they worked at or in health centres, school health, integration units, youth clinics, specialised psychiatric care, youth clubs and (re) engagement initiatives.

Young people were recruited with the help of professionals and gatekeepers at schools, youth centres and homes for unaccompanied minors. Covering diversity in terms of gender, ethnic background, sexual identity/orientation, and functionality, 63 youth aged 14–27 years participated in the study (29 young women, 33 young men and 1 not identifying as either or).

### Data collection

We used open-ended qualitative interviews, both individually and in groups, for data collection. In total, three of the authors (FJ, MC and MW) conducted 42 interviews between April–October 2018: 16 individual interviews with professionals and 26 FGDs, of which 11 were with professionals and 15 with youths. Table 2 provides site specific details. Since the number of sites and participants was not determined beforehand, we followed an emergent design by conducting interviews and FGDs until similar information started to emerge and until the team felt that enough information had been gathered to address the aim of our research project [19].

**Table 2:** Site specific details for data collection

	Youth	Professionals		
	Focus groups	Individual interviews	Focus groups	Total
**Site one (S1)**	1	5	1	7
**Site two (S2)**	5	3	3	11
**Site three (S3)**	3	2	2	7
**Site four (S4)**	2	–	3	5
**Site five (S5)**	4	6	2	12
**Total**	15	16	11	42

The interview guides (adapted for youth and professionals, respectively, see Supplementary material 1–2) related to youths’ situation regarding ‘life in the rural area’, ‘health situation’, ‘access to health care’, ‘collaboration between institutions’ ‘strategies for care and support’ and ‘suggestions for improvements to strengthen rural youths’ social/health situation’. One or two researchers from the team (FJ, MC and MW) conducted the individual interviews, while at least two of the researchers attended the FGDs with one acting as moderator and the other as co-moderator. The interviews lasted between 30 and 110 min (with an average of 60 min) and were conducted in either Swedish or English (with some professionals), digitally recorded and transcribed verbatim.

### Data analysis

The analysis started directly after each interview when the responsible researchers independently summarised their understandings. This means that data collection and analysis overlapped, so that the recruitment of new sites and participants followed insights gained from the preliminary analysis of the previously gathered data.

The framework ‘landscapes of care’ [23] was chosen in accordance with an emergent design, meaning that the initial analysis of the interviews guided the choice of theory. Abductive thematic analysis was used to analyse the data [34], which implied that during the analytical process we oscillated back and forth between the empirical material and the concepts. Specifically, while the analysis of the interviews guided us into the conceptual framework (data-driven), after the framework was chosen, the team went back to the material to further scrutinize the data using the concept ‘landscapes of care’ as an analytical lens (theory-driven).

In accordance with the steps of Braun and Clarke’s thematic approach [34], the analysis was conducted in the following way. Firstly, the transcripts were read to familiarise ourselves with the data and contribute with interpretations from various perspectives. Secondly, the transcripts were systematically coded. Thirdly, codes with similar content were clustered into preliminary themes. The fourth step involved comparisons of the emerging themes within and between the sites to identify central themes running across all sites. At the fifth step, we refined the specifics of each theme informed by our conceptual framework. Finally, through writing at the sixth step, each theme was further described and connected with the others to ‘tell the story of the data’ by linking data extracts with deeper argumentation in relation to our aim. The first steps (one to four) were ultimately data-driven followed by a more theory-driven phase (steps five to six) informed by our conceptual framework on ‘landscapes of care’ [23].

## Results and discussion

In the following combined results and discussion section, the themes developed are presented and discussed in connection with existing literature, and especially our conceptual framework, while being accompanied by quotations from the participants to illustrate and corroborate the content. Specifically, through analysing participating youths and professionals’ narratives, we developed one main theme, ‘landscapes of care and despair’ comprising the two themes: ‘(dis)connectedness’ and ‘extended support or troubling gaps’. In accordance with Milligan and Wiles [23], our results portray ‘landscapes of care’ as encompassing various health-promoting and enabling features spanning across structural and organisational levels while emphasising the importance of rural youth experiencing empathic encounters with people alongside senses of inclusion and belonging to place (see also [24]). At the same time, negative aspects were brought to the fore and by making them explicit, we expanded our framework to illustrate how landscapes can simultaneously be a source of despair, for rural youth in general and minority groups in particular. To ensure the participants’ confidentiality and anonymity, pseudonyms are used throughout the following text.

### (Dis)connectedness

Based on the idea that care encompasses people-place interactions and also interpersonal relations that occurs within formal and informal spaces outside traditionally medical settings [23, 24], this first theme, ‘(dis)connectedness’, portrays how various physical and social features of landscapes can give rise to both care and despair.

With reference to health-promoting features characterising landscapes of care, the participating youth discussed several aspects contributing to feelings of belonging, defined by Antonsich [38] as being rooted in or emotionally attached to a place. In this regard, their narratives painted a classical picture of rural areas as being marked by sparse settlements, tranquillity and safety [6]. These aspects appeared through positive expressions like “it’s generally calmer [here], less people and more free” (Tim, ninth grade student, S5) and “you feel really safe here and like, walk around knowing that nothing will happen” (Anna, high school student, S2). Adding to these representations of ‘peace and quiet’ [5], the youths valued the pristine nature through outdoor activities like snowmobiling and fishing that – especially in young men – seemed to bring meaning to their spare time and promote wellbeing. Besides being experienced through physical and more tangible features, attachments to their home place appeared to also be felt, as depicted by Lisa (high school student, S2): “it’s not as if I have any hatred for [the local area], I love it, and always think it’s so nice to come home when I’ve been away”. Following conceptions of the ‘rural idyll’, where Norwegian youth have portrayed peripheral areas as characterised, for example, by solidarity, neighbourliness and spirits of cooperation [6], the participants further described a closeness within their locale. This aspect came across through reports of tight-knit communities shaped by strong social ties where “everyone knows everyone” and people both friendly and helpful.

Besides expressing ‘idyllic’ accounts of affection for, senses of comfort in and feelings of connectedness to their locale, a negative story portraying landscapes of despair, was told in parallel. On the one hand, this emerged from a perceived lack of opportunities for organised leisure, education and employment, which in line with discourses on rural deficit and decline [4, 10] appeared as (re) constructions of rural areas as boring [5] and dull [6], with “nothing to do” and “nowhere to be”. On the other hand, this tale comprised reservations about the benefits of being close and visible to include descriptions about the problems of rural proximity and transparency. Following Haugen and Villa [39], this came across in the youths’ narratives as strict and limiting social norms with gossip and rumours appearing as obstacles to privacy, as sources of labelling and as control mechanisms that pressured youth to look, act and behave in certain ways.

*It’s just this trash-talk, I don’t even have to say anything … Say I have a fever and don’t have the energy to put make-up on, I haven’t had the energy of putting a pair of jeans but wear sweatpants to the store. Then you are … yes, my god what a chatter it will be. You cannot walk a certain way … In one way, I long for [a bigger city down south], where you can be yourself, it’s not a problem how you look. (Sophie, young adult, S3).*

In accordance with Sophie’s narration showing the struggles of conformity associated with intrusive aspects of informal social control, the youths’ discussions illustrated how “everyone has to be like everyone else” in order to ‘fit’ within landscapes more generally and peer groups in particular. Based on this notion, portrayed was several examples of racist accounts where discourses and practices that distinguished ‘us’ (the ones who belong in a place or to a group) from ‘them’ (the ones who do not) [38] appeared to create clear social and physical boundaries between native- and foreign-born refugee youth. This aspect became visible in the immigrating youths’ narratives, for example, through descriptions about the difficulties of getting integrated into and accepted by the community, no matter how hard you tried. From the perspective of native youth, in turn, it appeared through discussions portraying ‘them’ – the immigrating youth who could not speak the language or “behave” by following norms and laws – as different and inferior to ‘us’ who can. Adding to these discriminatory struggles, the youths’ believed that identifying and/or coming out as lesbian, gay, bisexual, transgender or queer (LGBTQ) might be difficult since it deviated from the heterosexual norm. In this regard, most participants assumed that youth with non-binary gender identities or non-heterosexual sexual orientations would (have to) leave for the cities to avoid the prejudice and isolation associated with being ‘different’ in the rural.

To this end, the theme ‘(dis)connectedness’ illustrates how circumstances of and in landscapes may be a source of care for some youth by allowing them to feel ‘at home’ in their rural locale and like they ‘fit’ within a group; circumstances that according to Antonsich [38] generate senses of connectedness to people and place on which belonging relies. Due to a perceived marginalisation and exclusion of different sub-groups of young people, the theme shows how conditions of and in landscapes might also be reason for despair, especially to minority youth. In line with Leyshon [40], the theme thus portrays how landscapes can be ‘enabling and inclusive’ as well as ‘restrictive and prohibitive’ at the same time, thereby contributing to complex landscapes characterised by care and despair.

### Extended support or troubling gaps

The second theme, ‘extended support or troubling gaps’, illustrates the importance of organisational and more formal arrangements in the provision and practices of care, while also suggesting that both political and affective aspects facilitate and hinder the process of caring *for* and *about* rural youth [23]. In particular, this theme describes how the mere existence of health and social services does not guarantee that youth will benefit from them, but rather that their access to care and experiences of being cared *for* and *about* involve both structural and interpersonal components.

Following Milligan and Wiles [23], who stress the value of ongoing commitments to and responsibilities for care, this theme captures features within landscapes that the participants’ saw as central to ensure good and consistent care for rural youth. In this regard, the participants considered the willingness of policy-makers and managers to prioritise youths’ needs and invest in their health as essential, for example, by providing staff at youth clubs and youth clinics with full time positions to increase the availability of services (see [41]). As further examples stressing the importance of caring at structural levels while taking long-term perspectives to care [31], emphasised was also the benefits of being able to work strategically, continuously and preventively with issues that mattered to youth. The professionals then further described the value of cooperating and communicating across sectors to care *for* youth, circumstances that appeared to be partly facilitated by the rural closeness and transparency.

Several examples of ‘pioneering’ care-work also appeared in the participants’ narratives, illustrating how care is largely an interpersonal concern involving ‘the provision of practical or emotional support’ ([23] p. 737). At the general level, this came across through descriptions of omniscient and hardy professionals that transcended barriers while extending their support beyond the roles and responsibilities of their profession to care *for* and *about* youth. More specifically, it comprised portrayals of practical assistances such as personalising and tailoring care according to need while “fixing appointments for them” (Elin, social councillor, S3) to help youth navigate within ‘complex, fragmented bureaucratic health systems’ ([42] p. 377). The work of pioneering professionals also involved accounts of empathy and compassion, with them stretching emotionally outside what might be expected in their profession. In line with Milligan and Wiles ([23] p. 738), who stress that ‘care-givers do not simply do things for people’ but support them with ‘personal attention’ and ‘encouragement’, this aspect came across, for example, through descriptions of professionals trying to help young people ‘at-risk’ by compensating attentively for parental shortcomings. From the youths’ perspectives, in turn, these emphatic and compassionate aspects of care appeared through descriptions of being treated as equals and of having someone – besides family and friends – that took interest in and cared *about* them.

*I got a great psychologist who took care of me. It was (...) we talked, and it was like five appointments set up at a time and I thought “finally, finally someone that sees me”. (Sophie, young adult, S3).*

Parallel to the above health-promoting or enabling features, the participants’ discussed several obstacles to the provision, practices and experiences of care. Here, the professionals described how financial constraints, problems with recruiting and retaining staff as well as collaboration challenges emerging from the different roles and responsibilities of actors made it difficult to prioritise youth and provide them with adequate care. From the youths perspectives, it appeared to result in ‘system inefficiencies’ ([43] p. 10) and troubling gaps in service delivery, with the care being shaped by long waiting times, needs to repeat their story and fragmented or disrupted contacts which implied that they rarely saw the same physician twice: “the craziest thing is that I’ve never had the same medical doctor and I’ve been going to the psychiatry [team] for over ten years” (Lexi, young adult, S3). In accord with Robards and colleagues’ [43] notions of an unresponsive rural health system for young people, the youths also typically considered various health services as vital, but not necessarily as providing appropriate care. This issue built on the perception that health centres often disregarded problems that went beyond the reason for consultation with a “you only get what you came for” attitude, while being mainly concerned with somatic problems which yielded tensions between what the youths’ wanted or needed and what they actually received.

*Alva: It is like... they sit there and just “we give you this medicine, it might work” and then when you come back and say that it doesn’t work, either they increase the dose or give you a new medicine. It’s all or nothing. You never get to talk about things.*

*Nina: They are just like*” *here, take this and maybe you feel better”.*

*Alva: Yes.*

*Nina: That they just don’t care about us, just like “never mind how you feel”. (Alva and Nina, ninth grade students, S1).*

As illustrated in Alva and Nina’s discussion, most youths’ wanted and needed someone that they could talk to and address face-to-face. This meant that although they recognised the importance of ‘care technologies’ to overcome rural problems of accessibility and anonymity, the expansion of eHealth was generally not considered to be an adequate or optimal solution since it allowed caring *for* to ‘become progressively more *dis*embodied’ ([23] p. 742).

In line with the construction of youth as a transitional period [44] and a ‘maturity gap’ in the life course [45] where young people are considered en route to, but not able to fully participate in, an ‘adult society’, the narratives illustrated how the challenges as well as the strengths of rural youth were often overlooked or disregarded in their community. Specifically, there was a strong general perception among the youths’ that local policy-makers were distant and degrading others that rarely saw, heard or acted on their needs, which came across in expressions like “I think it’s sad that politicians don’t think you have a voice that is important enough just because you’re not eighteen” (Erik, high school student, S3). The professionals largely corroborated this view of accountability [46] by explaining how young peoples’ opinions seldom were considered since older generations typically saw youth as ‘topics’ of rather than ‘actors’ in the community discussions. In addition, while some sites had formal structures for ensuring a democratic representation of young people through the youth councils, their role as “referral bodies” were generally questioned by the young people involved.

*Dani: In terms of democracy, we really didn’t get a response from the municipality when we tried to work politically. But with events, we could do as much stuff as we wanted.*

*Jonah: Yes, that is when the municipality started to react.*

*Dani: Yes, or rather it was like … instead of the youth council being a democratic body as it should be, it became a group of young people that created events for each other. And that is very practical, but then at the same time they [the politicians] increase the price of bus tickets and implemented both this and that without … then just writing, “no consequences for children and youth”, even though such things matter. (Dani and Jonah, young adults, S4).*

Instead of acting as pathways to ‘real’ influence, efforts within the youth councils were often consultative and reduced to the arrangement of events. This generated a lot of frustration among the youth involved while aligning with Coe and colleagues’ ([47] p. 1331) notions that social activities seem to be an ‘important but insufficient aspect of political action’.

Overall, the theme ‘extended support or troubling gaps’ indicates that the provision of care for rural youth should follow from the ‘priority of care’ [48] by moving beyond personal and practical aspects of service delivery to also include the political and the participatory. In line with Milligan and Wiles ([23] p. 747) notions ‘blurring of boundaries’, it further shows how the practices of care involve the pioneering work of committed and compassionate professionals who span across institutional spaces while stretching emotionally outside their profession to care *for* and *about* rural youth. Conversely, the theme illustrates how the care sometimes neither align with youth wishes nor correspond to their needs. This followed largely from experiences of receiving fixed rather than flexible services that focused on specific problems instead of seeing the whole person. Additionally, the theme show how many youth felt ignored (and sometimes even exploited) by seemingly ‘care-less’ policy-makers who delegated responsibility without allocating resources or power thereby preventing them from *really* participating in decision making to extend their influence beyond mere consultations and instrumental engagements.

### Study strengths and limitations

Our analysis comprised an extensive dataset including diverse sites regarding, for example, location, size and socio-economic situation; different youth voices in terms of, for example, ethnicity, gender, functionality, age and sexuality; and various vocations with professionals working across many sectors. Using a conceptual framework coupled with thematic analysis, we chose to illustrate the richness, diversity and discrepancies in the material by illustrating a multitude of ‘care-ful’ and ‘uncaring’ aspects and experiences. Along with Braun and Clarke’s [34] criteria for rigor, the data was thoroughly coded and analysed by scholars *active* in the research process. Trustworthiness was further achieved through prolonged engagement with the participants, which was aided by several personal contacts and site visits that allowed us to build trust and familiarise with the setting. The interdisciplinary research team contributed to critical assessments, triangulation and the integration of concepts.

Despite the diversity captured, there may be perspectives that we have not yet covered. In particular, although we tried to involve Sámi youth, capturing their experiences and perceptions was unfortunately not possible at the time of data collection.

## Concluding remarks

Through the narratives of our participants, the developed themes ‘(dis)connectedness’ and ‘extended support or troubling gaps’ align with Milligan and Wiles [23] conceptions regarding ‘landscapes of care’ while also expanding them to include more dissonant experiences of ‘despair’ as captured in the main theme.

Specifically, our findings comprise accounts of ‘care-ful’ landscapes characterised, for example, by youths’ connectedness and senses of belonging to people and place, individual and collective agency through the pioneering work professionals and civically engaged youth, as well as ongoing commitments and compassion where professionals saw, heard and responded consistently to young people’s needs. While the themes subsequently indicate that within the diverse group of youth some may be cared *for* and *about*, a negative story was told in parallel, indicating that landscapes may not be entirely or necessarily caring. This tale comprised various structural, organisational and interpersonal hardships that appeared to influence the youths, for example, through feelings of alienation and frustration. These experiences, which we conceptualised as despair, seemed to partly emerge through a lack of future prospects and recreational options; through their exclusion from policy arenas, public debates and peer groups; as well as through gaps in the provision and practices of care.

In summary, our study highlights the central role that care plays within different aspects of rural dwellers individual and collective lives, thus removing some shade from an otherwise marginalised concern [31]. The focus here on ‘dwellers’ rather than ‘youth’ is intentional, because we have depicted how care is not just handed to young people within certain landscapes, but rather co-produced through multidirectional dependencies between themselves and their environment [25]. Corresponding to representations of rural youth as ‘the others’ [49], our findings correspond to the work of Valentine [50], by showing how rural areas seem to be largely shaped by and constrained to adult expectations and institutions. However, while the marginalisation of youths may partly depend upon the power and overall ‘care-lessness’ of adults, young people appear to also alienate each other. This illustrates how youths often engage in intersecting and multi-layered processes of inclusion and exclusion [40] contributing, again, to complex and entangled ‘landscapes of care and despair’.

## Supplementary information

**Additional file 1.**

**Additional file 2.**

## Data Availability

The dataset analysed during the current study is not publicly available since it contains sensitive information but is available from the corresponding author on reasonable request.
