# Supplementary material for: Landscapes of care and despair for rural youth – a qualitative study in the northern Swedish ‘periphery’
Source: Int J Equity Health. 2020 Oct 2;19:171. doi: 10.1186/s12939-020-01288-z (PMC7531094; doi:10.1186/s12939-020-01288-z)
Supplement: Supplementary file 1 — Additional file 1. [file 12939_2020_1288_MOESM1_ESM.docx]

**Supplementary material 1
Interview guide – focus group discussions with youth**

**Introduction**

1. What is it like to live here in X?

**Health situation among youth**

1. How are youth feeling here in general?
2. What problems do youth here face?
3. What do they need to feel good and be healthy?

**Access to health and social services**

1. Where can you as a young person turn for help with the problems you just described?
2. How do you think young people experience these services? Regarding e.g. distance, staffing, costs, travel, opening hours, etc.
3. Do you feel that something is missing, or do young people get the help and support they need?
4. Are there particular groups of young people who do not get the help they need?
5. Do you think that the situation you describe would look different in a larger city? How?
6. Can you describe some advantages and disadvantages of living in a rural area?

**Collaboration**

1. How do you feel the collaboration between, for example, the school and the health center works?

**Strategies for care and support**

1. What is being done to manage or improve the situation you have just described?

**Suggestions for improvements**

1. How would you like to change this situation? What needs to be done?
2. What do you especially want us to take with us from this conversation?
3. Is there anything special that needs to be improved?

Thank you for participating in this focus group discussion, do you have any questions or comments?
