# Supplementary material for: Landscapes of care and despair for rural youth – a qualitative study in the northern Swedish ‘periphery’
Source: Int J Equity Health. 2020 Oct 2;19:171. doi: 10.1186/s12939-020-01288-z (PMC7531094; doi:10.1186/s12939-020-01288-z)
Supplement: Supplementary file 2 — Additional file 2. [file 12939_2020_1288_MOESM2_ESM.docx]

**Supplementary material 2**

**Interview guide – interviews with professionals**

**Introduction**

1. Can you describe your work to me? What are your tasks?

**Health situation among youth**

1. From your perspective, how are youth feeling here in general?
2. What problems do they face?
3. What do they need to feel good and be healthy?

**Access to health and social services**

***Generally in the municipality***

1. Where can youth turn for help with the problems you have just described?
2. How would you describe youth’s access to health and social services?
3. What services are available for youth? What services are not available?
4. What implications do you think a youth clinic would have (or what implications does it have)?
5. What role does the school and school health play?
6. Are there particular groups of youth who do not get the help they need?
7. Do you think that the situation you describe would look different in a larger city? How?

***Specifically within the service of the professional***

1. At your workplace, how do you work to meet the needs of youth?
2. How do you experience your ability to meet the needs of youth?
3. What aspects would make the work easier for you?

**Collaboration**

1. What actors are involved in collaborating around youth and youth-related issues?
2. What works well and how could the collaboration be improved?
3. How is the work affected by conditions (positive and negative) of rural areas?

**Strategies for care and support**

1. What is being done to manage or improve the situation you have just described?
2. How do you think youth experience these initiatives?
3. Any other innovative strategies to improve youths’ access to health and social services?
4. What strategies/programs exist (or are missing/need to be developed) to reach youth in vulnerable situations?

**Suggestions for improvements**

1. How would you like improve the situation of rural youth?
2. What do you especially want us to take with us from this conversation?
3. Is there anything special that needs to be improved?

Thank you for participating in this interview, do you have any questions or comments?
